# Sarcomatoid malignant pleural mesothelioma treated with nivolumab: A case series

**DOI:** 10.3892/ol.2022.13522

**Published:** 2022-09-22

**Authors:** Kentaro Hashimoto, Hiroaki Ozasa, Akihiko Yoshizawa, Hiroshi Yoshida, Tatsuya Ogimoto, Kazutaka Hosoya, Masatoshi Yamazoe, Hitomi Ajimizu, Tomoko Funazo, Hironori Yoshida, Yuichi Sakamori, Toyohiro Hirai

**Affiliations:** 1 Department of Respiratory Medicine, Graduate School of Medicine, Kyoto University, Kyoto-shi, Kyoto 606-8507, Japan; 2 Department of Diagnostic Pathology, Kyoto University Hospital, Kyoto-shi, Kyoto 606-8507, Japan

**Keywords:** PD-1 inhibitor, ICT, effectiveness, PD-L1 expression, immune-related adverse events

## Abstract

Immune checkpoint therapy (ICT) with nivolumab has been widely used to treat malignant pleural mesothelioma (MPM) since clinical trials confirmed its efficacy. However, only a few clinical trials have been conducted for the treatment of sarcomatoid MPM, which is a rare histological type of MPM. Additionally, clinical reports of sarcomatoid MPM are scarce. Therefore, the benefits and risks of nivolumab treatment for sarcomatoid MPM remain unclear. The present report describes the treatment of 3 cases of sarcomatoid MPM (all 3 were men) with nivolumab monotherapy. In all three cases, nivolumab was effective despite variations in the duration of treatment, although side effects were observed in 2 patients. Programmed death ligand 1 (PD-L1) expression was positive in all 3 cases. In particular, the patient with the highest PD-L1 expression had the most rapid response of the 3 patients, and the effect lasted as long as those of the other 2, despite receiving the smallest number of doses of nivolumab. It has been reported that sarcomatoid MPM tends to respond poorly to chemotherapy and express higher levels of PD-L1 than epithelial MPM; thus, ICT may be necessary in these cases. This case series suggests that ICT with nivolumab is a promising treatment option for sarcomatoid MPM.

## Introduction

Malignant pleural mesothelioma (MPM) is a malignant disease that is primarily caused by asbestos exposure and has a poor prognosis (1). MPM is pathologically classified into three types: epithelial, sarcomatoid (desmoplastic as a subtype), and biphasic (2). The sarcomatoid and biphasic types together are called the non-epithelial type and are rarer than the epithelial type. The prognoses for non-epithelial MPM are worse than that for the epithelial type because they respond poorly to existing cytotoxic chemotherapies, more effective treatments have been long awaited. Immune checkpoint therapy (ICT) with nivolumab is reported to be effective for the treatment of epithelial MPM [objective response rate (ORR), 29.4%; 2-year overall survival rate, 35.3%] and is expected to be a new treatment option for MPM (3). However, evidence for the treatment of non-epithelial MPM is scarce, and the combined results of the two existing clinical trials contained only 18 patients with non-epithelial MPM who received nivolumab (3,4). There are even fewer reports on the course of treatment in clinical practice; to the best of our knowledge, only a few cases have been reported (5,6). The collation of reliable evidence regarding ICT for non-epithelial MPMs is urgent. Here, we report three clinical cases of patients with sarcomatoid MPM (sMPM) treated with nivolumab at Kyoto University Hospital, Japan.

## Case report

### Case 1

**Figure 1.**
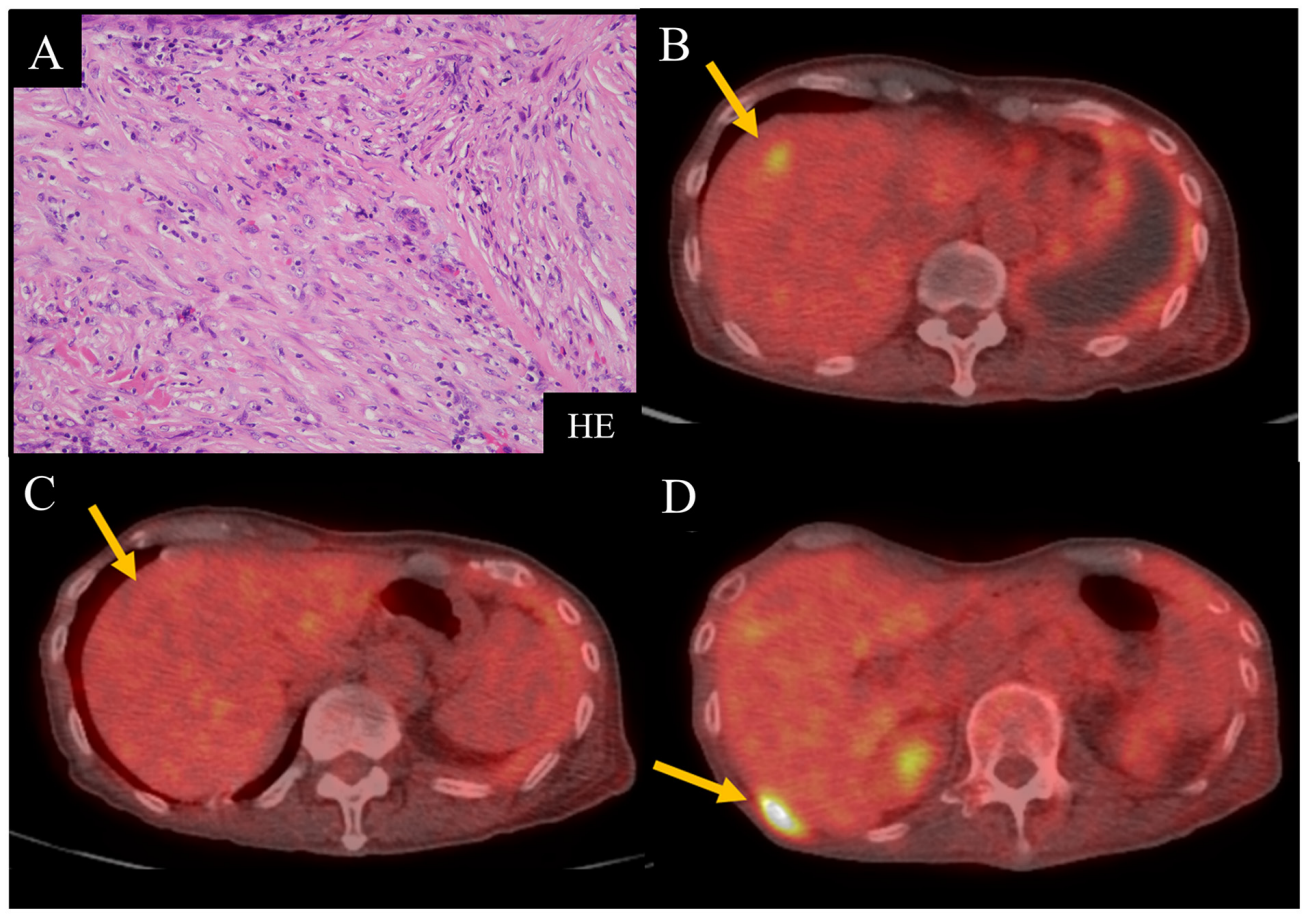
Pathological findings and FDG-based positron emission tomography/CT images of Case 1. (A) HE stain in surgical pleural biopsy (original magnification, ×40). (B) Case 1 before nivolumab treatment. The yellow arrow indicates FDG uptake in the liver metastasis. (C) FDG uptake in the liver metastasis (yellow arrow) decreased after five cycles. (D) Bone metastases (yellow arrow) subsequently developed. FDG, ^18^F-fluorodeoxyglucose; HE, hematoxylin and eosin.

A 73-year-old man was admitted to another hospital with left irregular pleural thickening (PT) and pleural effusion (PE). A surgical pleural biopsy was performed in that hospital. Histopathological findings showed atypical spindle cells distributed with inflammatory cells in a fibrous organization that was rich in collagen fibers, which invaded the striated muscle and adipose tissue (Fig. 1A). The patient was diagnosed with desmoplastic MPM. The patient was referred to Kyoto University Hospital for consideration of the multidisciplinary treatment since surgical treatment could not be performed at the referring hospital. However, the referring hospital is undisclosed in this report because this hospital is not affiliated with any of this study authors. After reviewing the case, we decided to treat the patient with systemic drug therapy. One cycle of systemic chemotherapy with carboplatin [area under the concentration-time curve (AUC)=5] and pemetrexed (400 mg/m^2^) (Carbo/Pem) was administered; however, the PT did not improve, and liver metastasis was confirmed. We deemed the Carbo/Pem treatment ineffective and initiated nivolumab as a second-line treatment. After five cycles of nivolumab, positron emission tomography (PET)/computed tomography (CT) using ^18^F-fluorodeoxyglucose (FDG) showed a reduction in liver metastases and a decrease in FDG uptake in the same area (Fig. 1B and C). Nivolumab treatment was judged to have achieved a partial response (PR) and was continued; however, new bone metastases appeared, and liver metastases reappeared after 12 cycles (Fig. 1D). Consequently, nivolumab treatment was judged to have resulted in progressive disease (PD) and discontinued. The patient was then treated with chemotherapy, but the disease worsened, and he died 4 months after completing nivolumab treatment. During the course of treatment, there were no side effects that could be attributed to nivolumab.

### Case 2

**Figure 2.**
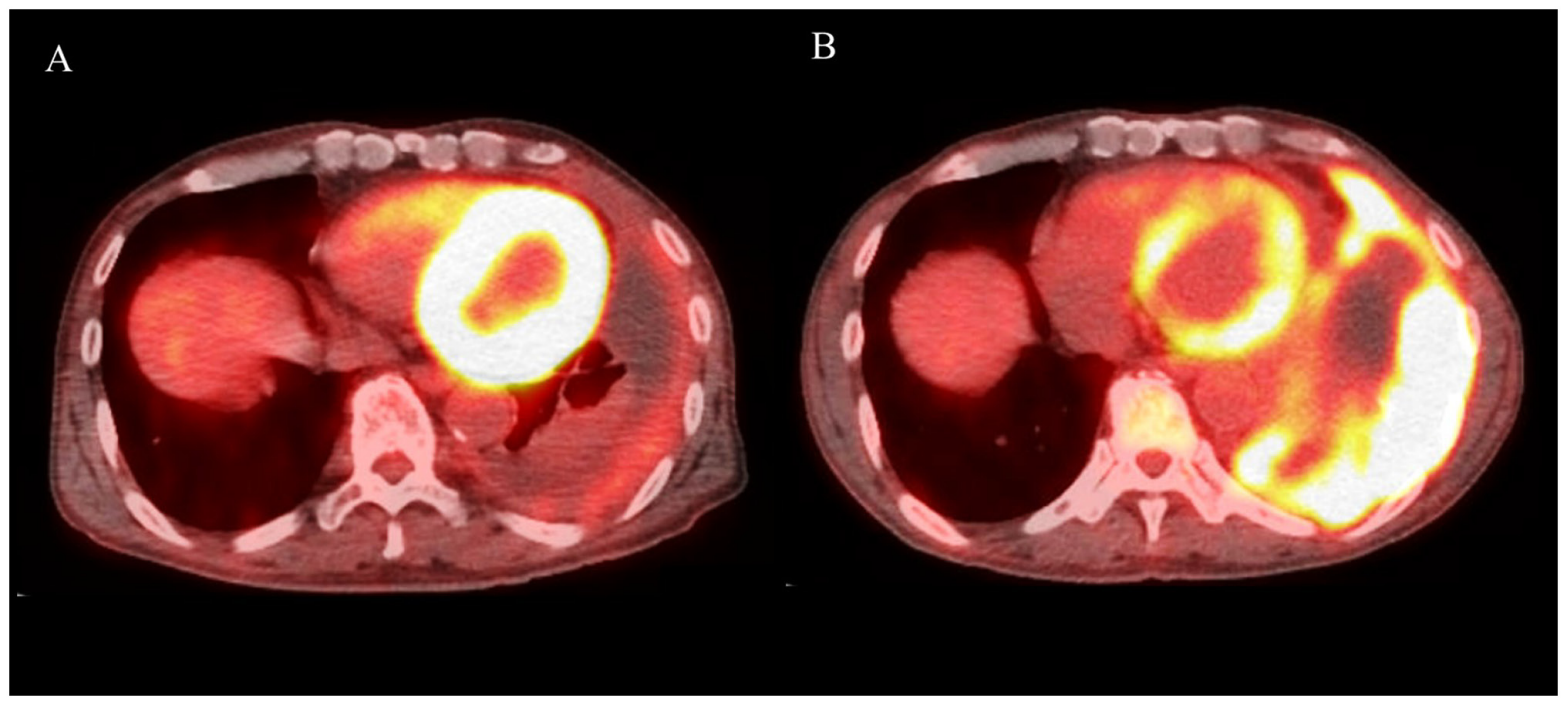
Positron emission tomography/CT images of Case 2. (A) After three cycles of nivolumab, the left pleural thickening and effusion improved, and ^18^F-fluorodeoxyglucose uptake decreased. (B) After nine cycles, the left pleural thickening and effusion worsened again. Other images are shown in our previous report (5).

**Figure 3.**
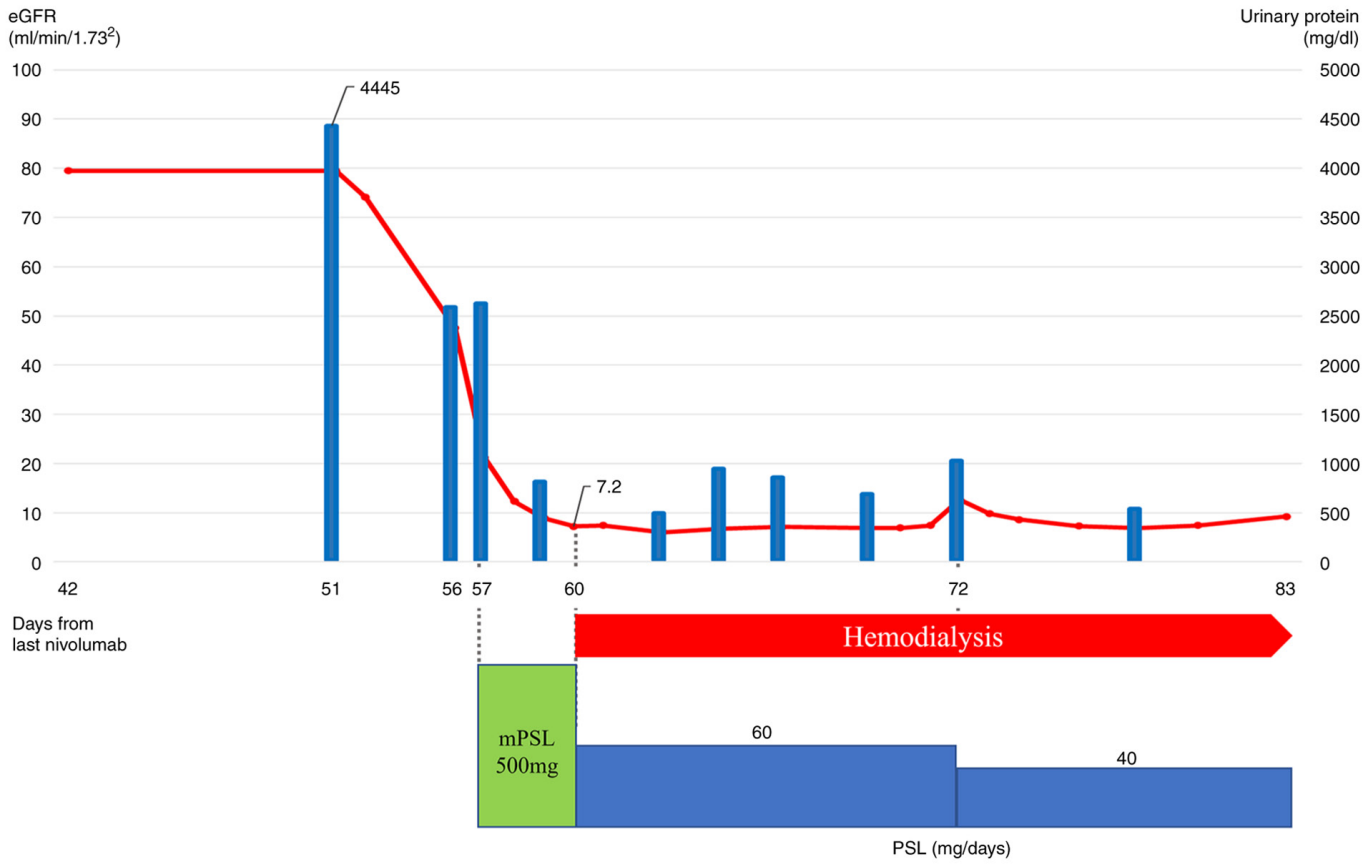
Clinical course of the adverse events that occurred in Case 2. The red line indicates the eGFR, and the blue bars indicate the urinary protein. On Day 51 after the last (9th) cycle of nivolumab, severe proteinuria was observed. Acute kidney injury occurred. Therefore, steroid therapy was started on Day 57 and dialysis on Day 60. eGFR, estimated glomerular filtration rate; mPSL, methylprednisolone; PSL, prednisolone.

A 66-year-old man was referred to our hospital with a large left-sided PE. PET/CT showed an irregular mass extending to the left pleura with increased FDG uptake. The patient was diagnosed with sarcomatoid or desmoplastic MPM by ultrasound-guided pleural biopsy. As the first-line treatment, Carbo (AUC=5)/Pem (400 mg/m^2^) was administered; however, after one cycle, the left PT worsened. Carbo/Pem was determined to be ineffective, and nivolumab was administered as the second-line treatment. After three cycles of nivolumab, PET-CT revealed a decrease in the left PT and PE, with a decrease in FDG uptake (Fig. 2A). In this case, the patient developed complicated eosinophilia and eosinophilic PE, which improved over time after nivolumab treatment. Further details on the progress of this case were previously reported (5). Then, we determined that nivolumab produced a good PR and continued the treatment; however, after nine cycles, the left pleural lesion thickening re-occurred with increased FDG uptake (Fig. 2B). We considered that the nivolumab treatment had resulted in PD and therefore discontinued it. Two months after treatment completion, the patient developed acute kidney injury and nephrotic syndrome due to immune-related adverse events (Fig. 3, baseline data as Table SI). Steroid therapy was started with dialysis, and although the patient showed signs of recovery, he later developed gastrointestinal bleeding and died. The autopsy revealed that the bleeding was due to metastasis to the small intestine. Here, we have reported Case 2 again to additionally describe the occurrence of side effects after the previous publication (5) and to make conclusions on the treatment choice as part of a case series that included the other two cases.

### Case 3

**Figure 4.**
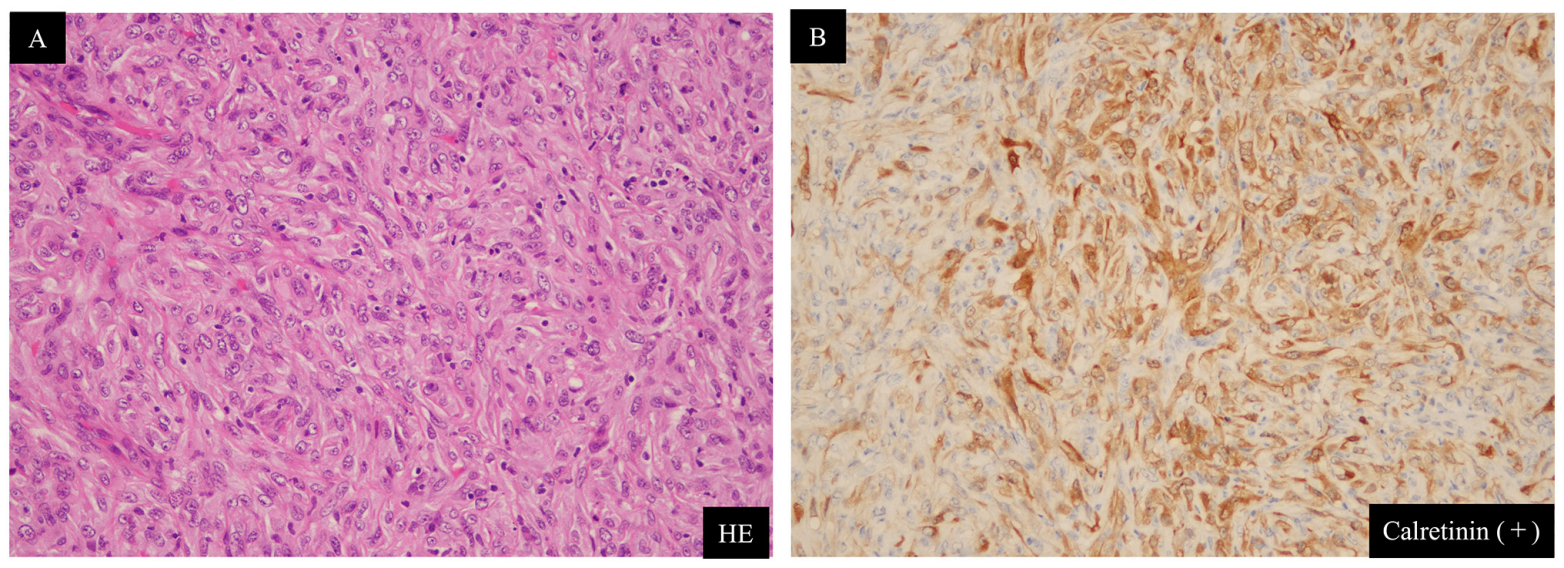
Pathological findings in Case 3 surgical pleural biopsy (original magnification, ×40). (A) HE stain. (B) Calretinin. HE, hematoxylin and eosin.

**Figure 5.**
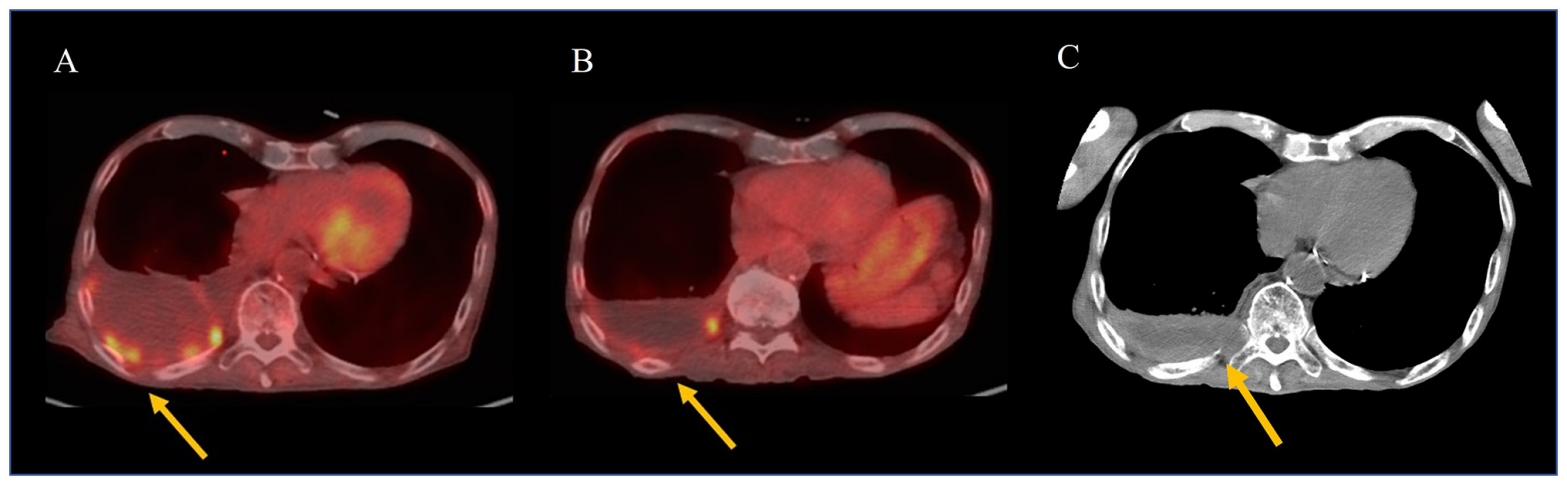
(A and B) Positron emission tomography/CT and (C) CT images of Case 3. (A) Case 3 before nivolumab treatment. The yellow arrow indicates FDG uptake in the pleural thickening. (B) After two cycles, the right pleural thickening and effusion (yellow arrow) improved, and FDG uptake decreased. (C) At 6 months after the end of treatment, the pleural thickening worsened again (yellow arrow), and invasion of the pleura and ribs was observed. FDG, ^18^F-fluorodeoxyglucose.

**Figure 6.**
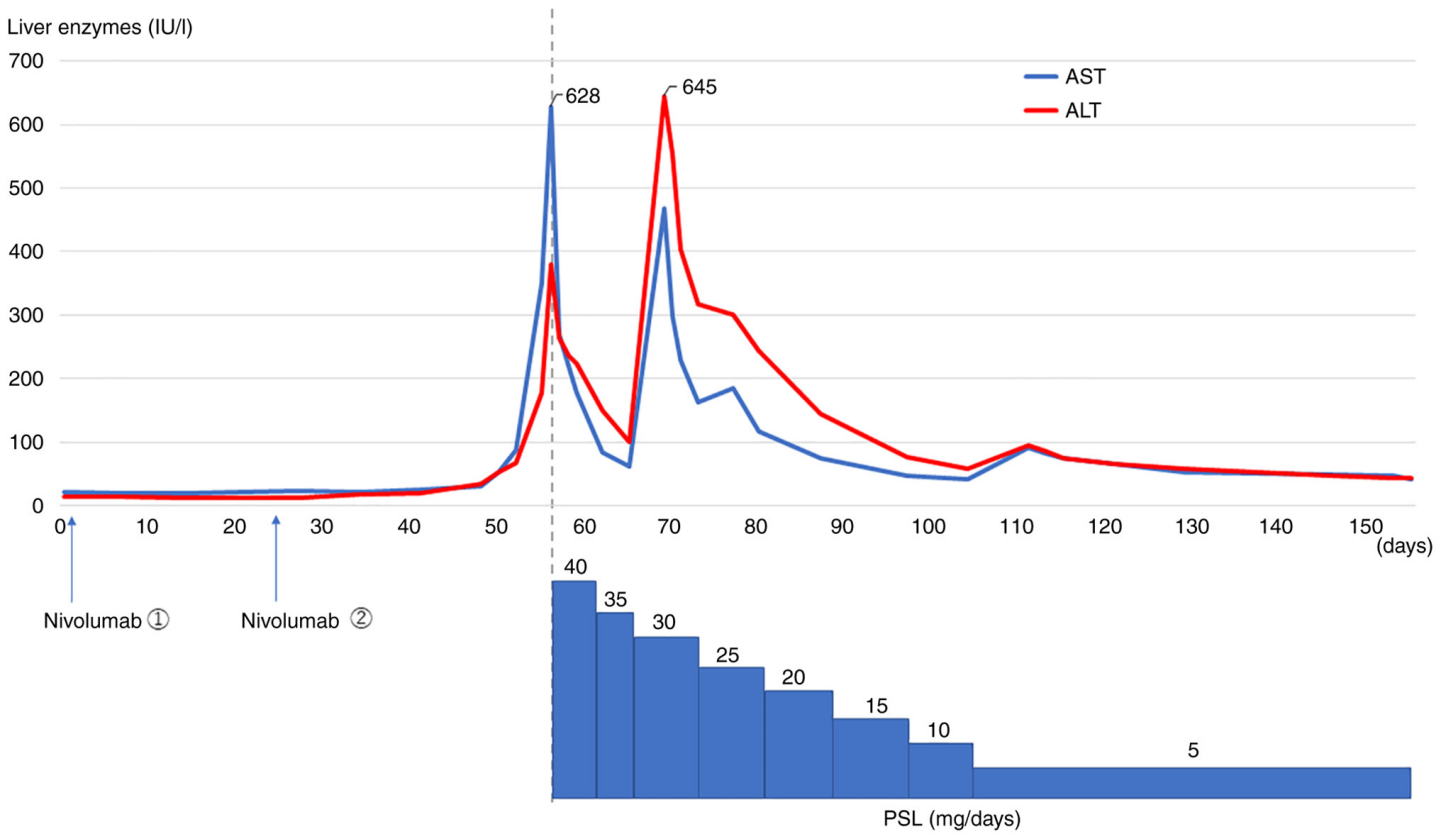
Clinical course in terms of the adverse events that occurred in Case 3. After the first cycle (Day 1) and second cycle (Day 28) of nivolumab administration, liver injury gradually developed from Day 50. On Day 56, the patient was judged to have severe liver injury, and steroid therapy was initiated. After the initiation of steroids, the liver injury gradually improved, and the hepatic enzymes were normalized by Day 120. ALT, alanine aminotransferase; AST, aspartate aminotransferase; PSL, prednisolone.

An 82-year-old man was admitted to our hospital with right PT and PE. Surgical pleural biopsy was performed. Histopathological findings showed atypical cells with large oval or spindle nuclei multiplying into a seat form with necrosis. These tumor cells were immunoreactive to calretinin but not to TTF-1 or p40 (Fig. 4). Based on these findings, the patient was diagnosed with sMPM. He was also diagnosed with neoplastic fever before biopsy. The patient was started on Carbo (AUC=4.5)/Pem (375 mg/m^2^), but the fever persisted along with high levels of C-reactive protein, and his performance status declined. After one cycle, no improvement was observed on the chest radiograph. Carbo/Pem was determined to be ineffective, and nivolumab was administered as the second-line treatment. After two cycles of nivolumab, the fever resolved and PET/CT showed a decrease in the right PT and PE, as well as a decrease in FDG uptake (Fig. 5A and B). However, before the start of the third cycle, the patient developed liver dysfunction due to immune-related adverse events (Fig. 6, baseline data as Table SII). Although the liver damage improved with steroid treatment, nivolumab treatment was discontinued because of the adverse event. Thereafter, the patient was placed on a treatment-free follow-up, and the tumor remained stable for 6 months. The patient died of aspiration pneumonia 1 month after the tumor began to re-grow (Fig. 5C).

**Figure 7.**
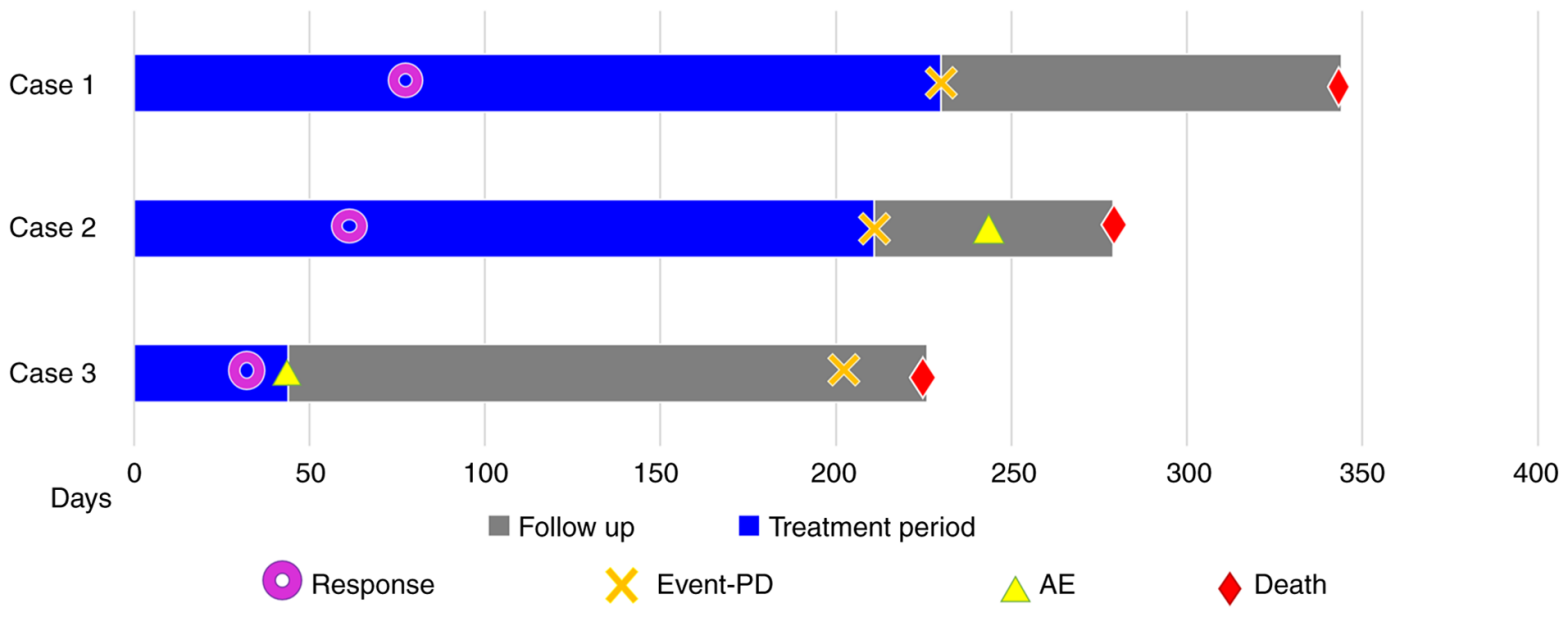
Clinical course of the 3 cases shown using the swimmer's plot. Tumor response was observed in all the patients. The mean time to response to nivolumab therapy was 58 days. Furthermore, 2 of the 3 patients experienced adverse effects, with a time to onset of symptoms of 243 and 43 days, respectively. AE, adverse event; Event-PD, event-progressive disease.

**Figure 8.**
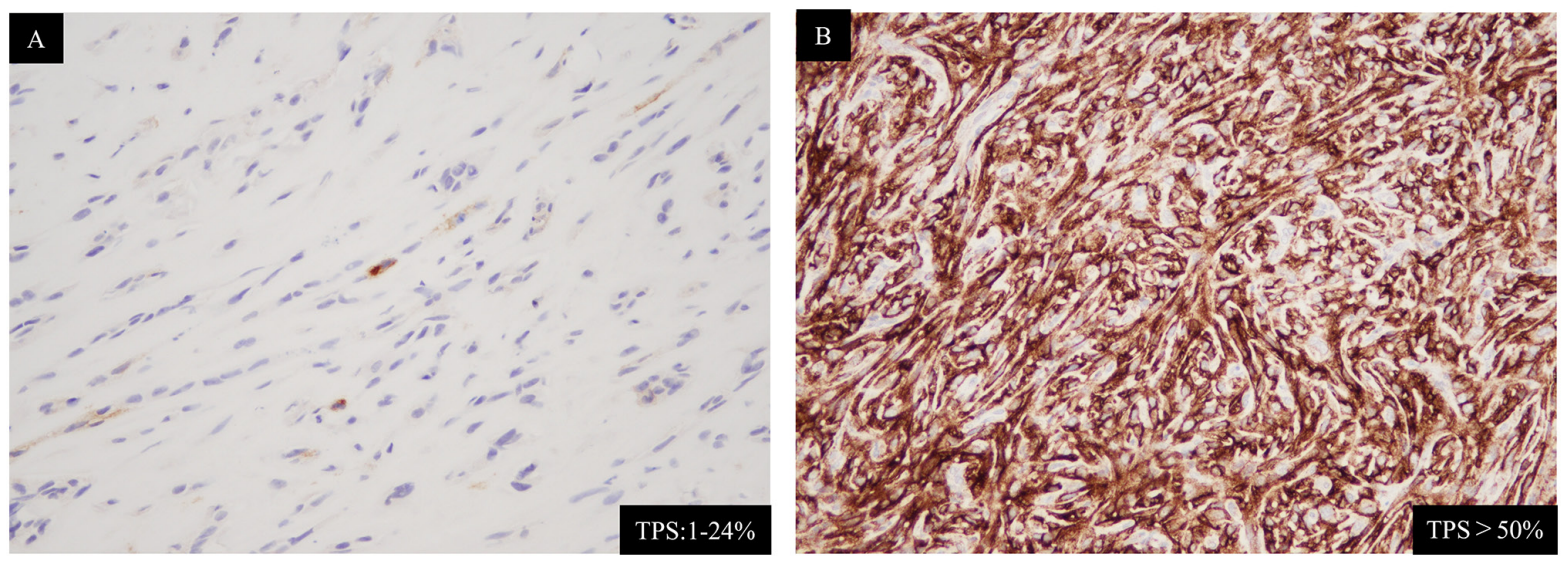
Immunohistochemistry of programmed death ligand 1 protein in the tumors of Cases 2 and 3 (using the DAKO 22C3 TPS method). The result for Case 1 was not in our hospital records. (A) Case 2. TPS, 1–24%. (B) Case 3 (TPS, >50%). TPS, tumor proportion score.

The clinical characteristics of the three patients are summarized in Table I. Fig. 7 shows the progress of the patients after starting nivolumab therapy. The three patients had varied histories of smoking, and two of them had a history of known asbestos exposure. Notably, the time to disease progression for the three cases following nivolumab treatment was 223, 211, and 202 days, respectively, which is similar to the median progression-free survival reported in the two previous trials (3,4), despite the difference in the number of cycles of nivolumab administration among the three patients, with 12, 9, and 2 cycles, respectively. We tested the expression of programmed death ligand 1 (PD-L1) in tumor cells, using DAKO 22C3 tumor proportion scoring method (Fig. 8). The results were positive in all the three cases, with Case 3 having more than 50% PD-L1 positivity (Cases 1 and 2 were 1–24% positive).

## Discussion

Nivolumab showed some efficacy against sMPM in the three cases treated at our hospital. In a previous study on the use of nivolumab to treat MPM (the MERIT study), three cases of sMPM were included. Nivolumab was reported to be effective in two of these cases, suggesting that the treatment may be more effective in sarcomatoid than in epithelial MPM (3). Historically, sMPM has been less likely than epithelial MPM to respond to cytotoxic chemotherapy (7). Thus, immunotherapy is expected to become an increasingly important treatment, particularly for sMPM.

In this study, we report three cases of sMPM in which the tumor cells tested positive for PD-L1 expression. In non-small cell lung cancer (NSCLC) tumors, PD-L1 expression is known to be a biomarker of the therapeutic efficacy of anti-programmed cell death protein 1 (PD-1) inhibitors (8). In MPM, high expression levels of PD-L1 have been associated with non-epithelial histology and poor prognosis (9–11). However, these results were reported before the introduction of ICT. Long-term follow-up data from the MERIT study showed that PD-L1-positive tumors tended to have a higher ORR to nivolumab in patients with MPM (12). In the same report, progression-free survival and overall survival tended to be better for patients with non-epithelial tumors compared to those with epithelial tumors. Currently, PD-L1 expression is not recognized as a biomarker for predicting the efficacy of PD-1 inhibitors in MPM. However, based on this evidence, sMPMs may be more likely to have higher PD-L1 expression levels than epithelial MPMs and may benefit from ICT.

Recently, the combination of nivolumab and ipilimumab (Nivo/Ipi), an anti-cytotoxic T-lymphocyte-associated protein 4 inhibitor, became available for the treatment of various carcinomas. In NSCLC, the effectiveness of Nivo/Ipi has been shown to be almost equal, regardless of PD-L1 expression (13). However, there are concerns regarding the toxicity of Nivo/Ipi. Although manageable, the toxicity profile was less favorable than that of nivolumab monotherapy. For NSCLC with high PD-L1 expression, there is no consensus on the benefit of Nivo/Ipi over anti-PD-1 monotherapy. Therefore, anti-PD-1 monotherapy will continue to be a viable, less toxic, and generally effective option for NSCLC with high expression levels of PD-L1. Nivo/Ipi has also been reported to be effective against MPM, for which it has shown greater efficacy than systemic chemotherapy, including platinum-based agents, as a first-line treatment (14). This evidence indicates that the use of ICT in the treatment of MPM is expected to become increasingly important, and Nivo/Ipi will play a leading role. However, the side effect concerns are similar to those for NSCLC. There is a lack of evidence on which ICT treatment strategy should be used in sMPM, as is expected to respond to PD-1 inhibitors alone. We believe that this report is significant because it contributes to the body of knowledge on nivolumab treatment for sMPM, on which few reports exist. Yet, this study included only three cases from a single institution, which potentially limits the validity of our findings. For a more reliable report, it is necessary to gather similar cases from multiple centers and study them in more detail.

In conclusion, we described three cases of nivolumab treatment for sMPM, which has rarely been reported before. PD-1 monotherapy may be more effective in treating sMPM than it is in treating epithelial MPM, and nivolumab treatment is a promising treatment option.

## Supplementary Material

Supporting Data

## Figures and Tables

**Table I.** Summary of the clinical characteristics of the 3 cases.

Variable	Case 1	Case 2	Case 3
Age, years^a^	73	66	82
Sex	Male	Male	Male
Smoking, p-y	4	58	None
Asbestos	Yes	Uncertain	Yes
Histopathological diagnosis	Desmoplastic	Sarcomatoid/desmoplastic	Sarcomatoid
Stage (UICC ver. 8)	cT3N0M0 stage1B	cT3N0M0 stage1B	cT4N0M0 stage3B
Pre-treatment	Carbo/Pem	Carbo/Pem	Carbo/Pem
PD-L1 TPS (22C3), %	1-24	1-24	>50
Effect	PR	PR	PR
Time to response, days	83	57	34
Nivolumab cycles, n	12	9	2
Time to progression, days	223	211	202
Overall survival, days	344	279	226

a The mean age of the 3 patients was 73.7 years. p-y, pack-year; UICC, Union for International Cancer Control; Carbo/Pem, carboplatin and pemetrexed; PD-L1, programmed death ligand 1; TPS, tumor proportion score; PR, partial response.

## Data Availability

The datasets used and/or analyzed during the current study are available from the corresponding author on reasonable request.

## Abbreviations

AUC: area under the concentration-time curve
Carbo/Pem: carboplatin and pemetrexed
FDG: ^18^F-fluorodeoxyglucose
ICT: immune checkpoint therapy
MPM: malignant pleural mesothelioma
Nivo/Ipi: nivolumab and ipilimumab
NSCLC: non-small cell lung cancer
ORR: objective response rate
PD: progressive disease
PD-1: programmed cell death protein 1
PD-L1: programmed death ligand 1
PE: pleural effusion
PET: positron emission tomography
PR: partial response
PT: pleural thickening
sMPM: sarcomatoid MPM
TTF-1: thyroid transcription factor-1
