# Supplementary material for: Sarcomatoid malignant pleural mesothelioma treated with nivolumab: A case series
Source: Oncol Lett. 2022 Sep 22;24(5):402. doi: 10.3892/ol.2022.13522 (PMC9533663; doi:10.3892/ol.2022.13522)
Supplement: Supporting Data [file Supplementary_Data.pdf]

Table SI. Baseline safety data for Case 2 before the start of nivolumab treatment.

Variable	Value
WBC, x10^3/µl	10.65
Neutrophil, %	85.7
Lymphocyte, %	6.8
Monocyte, %	5.6
Eosinophil, %	1.7
Basophil, %	0.2
RBC, x10^4/µl	3.58
Hemoglobin, g/dl	10.3
Hematocrit, %	32.2
Blood platelets, x10^4/µl	399
AST, U/l	7
ALT, U/l	6
LDH, U/l	238
ALP, U/l	266
γ-GT, U/l	33
Total protein, g/dl	6.9
Albumin, g/dl	3.2
Total bilirubin, mg/dl	0.3
Creatinine, mg/dl	0.75
eGFR, ml/min/1.73^2	79.5
BUN, mg/dl	12

Creatine kinase, U/l	36
Na, mmol/l	137
K, mmol/l	4.8
Cl, mmol/l	101
Ca, mg/dl	8.4
CRP, mg/dl	5.9
Plasma glucose, mg/dl	130
Rheumatoid factor, U/ml	<8.0
IgG, mg/dl	876
IgA, mg/dl	175
IgM, mg/dl	57
KL-6, U/ml	133
TSH, U/ml	2.63
Free T4, ng/dl	1.18
Free T3, pg/ml	2.28
ANA, n	40
Homogeneous, n	40
Speckled, n	40
Nucleolar, n	N.D.
Peripheral, n	N.D.
Discrete speckled, n	N.D.
Cytoplasmic, n	N.D.
Others, n	N.D.

Urine creatinine, mg/dl	80
Urine UN, mg/dl	423
Urine glucose, mg/dl	<10
Urine protein, mg/dl	13
Urine NAG, U/l	37.1

WBC, white blood cells; RBC, red blood cells; AST, aspartate aminotransferase; ALT, alanine aminotransferase; LDH, lactate dehydrogenase; ALP, alkaline phosphatase; γ-GT, γ -glutamyl transpeptidase; eGFR, estimated glomerular filtration rate; BUN, blood urea nitrogen; CRP, C-reactive protein; KL-6, sialylated carbohydrate antigen KL-6; TSH, thyroid stimulating hormone; ANA, antinuclear antibody; NAG, N-acetyl-β-D-glucosaminidase; N.D., not detected.

Table SII. Baseline safety data for Case 3 before the start of the nivolumab treatment.

Variable	Value
WBC, $x10^3/\mu l$	5.46
Neutrophil, %	74.7
Lymphocyte, %	9.9
Monocyte, %	12.5
Eosinophil, %	2.7
Basophil, %	0.2
RBC, $x10^4/\mu l$	2.44
Hemoglobin, g/dl	7.8
Hematocrit, %	23.3
Blood platelets, $x10^4/\mu l$	128
AST, U/l	21
ALT, U/l	14
LDH, U/l	150
ALP, U/l	51
γ-GT, U/l	26
Total protein, g/dl	5.4
Albumin, g/dl	1.9
Total bilirubin, mg/dl	0.3
Creatinine, mg/dl	0.50
eGFR, $ml/min/1.73^2$	116.5
BUN, mg/dl	10

Creatine kinase, U/l	15
Na, mmol/l	139
K, mmol/l	4.4
Cl, mmol/l	102
Ca, mg/dl	7.8
CRP, mg/dl	11.0
Plasma glucose, mg/dl	94
Rheumatoid factor, U/ml	13.6
IgG, mg/dl	1119
IgA, mg/dl	336
IgM, mg/dl	34
KL-6, U/ml	181
TSH, U/ml	2.38
Free T4, ng/dl	0.918
Free T3, pg/ml	1.14
HBsAg, U/ml	<0.005
HBsAg test result	Negative
HBsAb, U/ml	90.5
HBsAb test result	Positive
HBcAb, C.O.I	7.5
HBcAb test result	Positive
HBV-DNA, log U/ml	<1.0
HCVAb, C.O.I	<1.0

HCVAb test result	Negative
HCV-RNA, log U/ml	<1.2
EBV-VCA Ab (IgG), n	160
EBV-VCA Ab (IgM), n	<10
CMV antigen test result (C10/C11)	Negative
C10 positive cells	0
C11 positive cells	0
ANA, n	40
Homogeneous, n	40
Speckled, n	N.D.
Nucleolar, n	N.D.
Peripheral, n	N.D.
Discrete speckled, n	N.D.
Cytoplasmic, n	N.D.
Others, n	N.D.

WBC, white blood cells; RBC, red blood cells; AST, aspartate aminotransferase; ALT, alanine aminotransferase; LDH, lactate dehydrogenase; ALP, alkaline phosphatase; γ-GT, γ-glutamyl transpeptidase; eGFR, estimated glomerular filtration rate; BUN, blood urea nitrogen; CRP, C-reactive protein; KL-6, sialylated carbohydrate antigen KL-6; TSH, thyroid stimulating hormone; HBsAg, hepatitis B surface antigen; HBcAb, hepatitis B core antibody; HBV, hepatitis B virus; HCVAb, hepatitis C virus antibody; C.O.I., cut off index; N.D., not detected; EBV-VCA Ab, Epstein-Barr virus-viral capsid antigen antibody; CMV, cytomegalovirus; C10, cytomegalovirus low matrix phosphoprotein 65 antibody C10; C11, cytomegalovirus low matrix phosphoprotein 65 antibody C11; ANA, antinuclear antibody.
